# Pediatric Absence Status Epilepticus: Prolonged Altered Mental Status in an 8-Year-Old Boy

**DOI:** 10.1155/2016/9238310

**Published:** 2016-11-16

**Authors:** Scott J. Adams, Melody Wong, Tahereh Haji, Shahmir Sohail, Salah Almubarak

**Affiliations:** ^1^College of Medicine, University of Saskatchewan, Saskatoon, SK, Canada; ^2^Division of Pediatric Neurology, Department of Pediatrics, University of Saskatchewan, Saskatoon, SK, Canada

## Abstract

Absence status epilepticus is characterized by a prolonged state of impaired consciousness or altered sensorium with generalized electroencephalographic abnormalities. It is most commonly diagnosed in patients with known idiopathic generalized epilepsy; however, it may also be the first presentation of epilepsy. Due to the subtle and variable manifestations of the condition, absence status epilepticus may be underrecognized, particularly in children. We present the case of an 8-year-old boy who experienced two episodes of prolonged altered mental status, subsequently determined to be absence status epilepticus with idiopathic generalized epilepsy with phantom absences. We discuss the classification, pathophysiology, clinical presentation, and electroencephalographic findings of pediatric absence status epilepticus and provide a practical overview for management.

## 1. Introduction

Absence status epilepticus (ASE) is characterized by a prolonged state of impaired consciousness or altered sensorium. It is most commonly diagnosed in patients with known idiopathic generalized epilepsy; typical absence status epilepticus is observed most frequently in patients with juvenile absence epilepsy, eyelid myoclonias with absence, perioral myoclonia with absences, and idiopathic generalized epilepsy with phantom absences [1]. However, ASE may also be the first presentation of epilepsy, and ASE may be underrecognized, particularly in children. Because the clinical presentation of ASE can be variable and subtle, proper and timely diagnosis of ASE can be difficult to achieve, and electroencephalography is critical to establishing a diagnosis. We present a case of ASE in a child who presented with two episodes of prolonged altered mental status, subsequently determined to be absence status epilepticus with idiopathic generalized epilepsy with phantom absences.

## 2. Case Presentation

An 8-year-old boy presented at our pediatric epilepsy clinic for assessment of episodes of altered mental status which first occurred three months earlier. The parents reported that the patient had slow responsiveness in the morning and was sluggish and mildly confused. Though he was able to attend school that day, the patient's teachers noted that his speech was not wholly comprehensible; he failed to follow instructions and had periods of forgetfulness. He continued to ambulate sluggishly throughout the episode, and bilateral ptosis was noted. The patient returned to baseline without intervention after approximately four hours.

**Figure 1:**
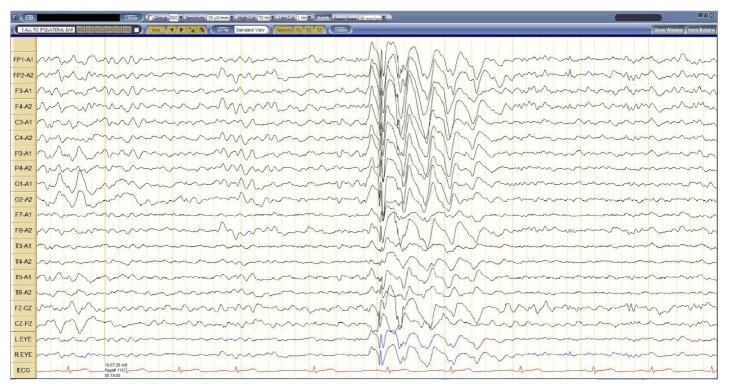
An interictal EEG sample showed 3 Hz generalized spike-and-wave discharges using an ipsilateral ear referential montage (sensitivity 15 *μ*V/mm; high frequency filter 70 Hz; low frequency filter 1 Hz; paper speed 30 mm/sec).

During the following three months, the patient's parents noted occasions of decreased rate of speech and the onset of staring spells. Each staring episode lasted a few seconds and was of sudden onset and offset without any postevent confusion. Past medical history revealed that the patient had two febrile seizures at the age of 15 months. Family history revealed that the patient's brother had a history of juvenile absence epilepsy. The neurological examination and brain MRI were normal. EEG showed 3-4 Hz spike-and-slow-wave discharges as well as polyspike-and-slow-wave discharges, consistent with idiopathic generalized epilepsy (Figure 1). Ethosuximide 250 mg twice a day was initiated for seizure prophylaxis, following which the patient's staring spells resolved.

One month after the initiation of ethosuximide, the patient presented to the emergency department upon experiencing a second episode of prolonged altered mental status. The patient was successfully treated with intravenous lorazepam three hours after the onset of symptoms and recovered immediately after receiving the injection. Ethosuximide doses were subsequently increased to 250 mg in the morning, 250 mg in the afternoon, and 500 mg at night. At the present time, the patient continues this regimen without any further seizures or adverse medication effects.

## 3. Discussion

Nonconvulsive status epilepticus (NCSE) is a type of seizure characterized by an alteration in cognition, memory, arousal, affect, motor learning, or motor behavior of at least 10–30 minutes in duration in the absence of tonic or clonic activity [2, 3]. NCSE can be diagnosed in both comatose or noncomatose patients and may be generalized, focal, or autonomic in origin. Classification of status epilepticus without prominent motor symptoms [3] is as follows:

1. NCSE with coma (including so-called “subtle” SE)
2. NCSE without coma
  (2.1) Generalized
    (2.1.1) Typical absence status
    (2.1.2) Atypical absence status
    (2.1.3) Myoclonic absence status
  (2.2) Focal
    (2.2.1) Without impairment of consciousness (aura continua, with autonomic, sensory, visual, olfactory, gustatory, emotional/psychic/experiential, or auditory symptoms)
    (2.2.2) Aphasic status
    (2.2.3) With impaired consciousness
  (2.3) Unknown whether focal or generalized
    (2.3.1) Autonomic SE

Typical absence status epilepticus (ASE) is observed in 10–30% of cases of idiopathic generalized epilepsy with absences [4], most frequently in patients with juvenile absence epilepsy, eyelid myoclonias with absence, perioral myoclonia with absences, and idiopathic generalized epilepsy with phantom absences [1]. Idiopathic generalized epilepsy with phantom absences was first reported in a child by Panayiotopoulos et al. [5]; however, the syndrome is not yet recognized by the International League Against Epilepsy [6] and further case reports and series are warranted to better understand this syndrome, which appears to be a variant of absence epilepsy, in pediatrics. Distinction must be made between ASE (absence status epilepticus) and absence status epilepsy, which is a separate condition proposed by Genton et al. in 2008, characterized by recurrent unprovoked periods of absence status with infrequent generalized tonic-clonic seizures, infrequent typical absences, and onset after puberty or in early adulthood [1]. Atypical ASE is observed in patients with symptomatic or possibly symptomatic generalized epilepsy such as Lennox-Gastaut syndrome [1]. Myoclonic status epilepticus, a generalized seizure characterized by continuous myoclonias of cortical origin, may be observed in nonprogressive encephalopathies such as Angelman syndrome [7]. NCSE may also present with primarily autonomic symptoms—autonomic status epilepticus—or might be of focal origin—complex partial status epilepticus (CPSE) [3]. Nonepileptic etiologies which should be considered in the differential diagnosis include head trauma, raised intracranial pressure, encephalitis, intoxication, metabolic derangements (such as medium-chain acyl-CoA dehydrogenase deficiency or hypoglycemia), and stroke [7].

Patients with ASE will typically present as seemingly aware, but with prolonged altered mental status and confusion. Rhythmic blinking, clonic twitching, automatisms, and myoclonic facial jerking may be variably present. Speech and the ability to perform tasks may also be affected depending on severity. Prodromal or postictal signs are rarely associated with ASE, and patients typically present with no focal neurological abnormalities and normal neuroimaging. Seizures begin and end abruptly and may last as long as several days [4, 7–10]. In its 2015 report, the International League Against Epilepsy (ILAE) suggested that absence seizures lasting a minimum of 10–15 minutes are likely to lead to prolonged and continuous seizure activity, though there is limited evidence for this definition [3].

EEG is a standard requirement for the confirmation of an ASE diagnosis. In typical ASE, EEG recordings indicate predominantly anterior, generalized continuous, waxing and waning, and rhythmic 3-4 Hz spikes, as well as polyspike-and-slow-wave discharges, usually with normal background activity [2]. Care must be taken to differentiate ASE from CPSE, as the latter may evolve from focal into generalized spike/sharp wave and/or suppressed wave discharges, usually with frontal predominance and slow, generalized background activity [2, 7]. As CPSE is associated with serious morbidity and mortality, differentiating ASE from secondarily generalized CPSE using interictal EEG is critical. EEG patterns of atypical ASE—reviewed in a 2012 compendium [2]—vary depending on the underlying epilepsy syndrome or encephalopathy and do not lend themselves to simplified criteria.

The pathophysiology of typical ASE is hypothesized to involve the failed termination of sustained, highly synchronized abnormal oscillatory rhythms in thalamocortical networks. Increased levels of gamma-aminobutyric acid (GABA) have been implicated in the pathophysiology of ASE, as hyperpolarization of thalamic relay neurons by GABA_B_ receptors can enhance oscillatory thalamocortical activity. This is consistent with reports which have suggested that antiepileptic drugs (AEDs) which increase GABA concentrations, such as vigabatrin (VGB) or tiagabine (TGB), may worsen ASE [11, 12].

Studies have suggested that carbamazepine (CBZ) and phenytoin (PHT) may also precipitate or worsen ASE [11–13]. This paradoxical effect may be explained by the higher probability of voltage-gated sodium channel state alignment in the thalamocortical area in those predisposed to absence seizures: dose-dependent inactivation of sodium channels by CBZ and PHT further increases sodium channel state alignment, potentially leading to neuronal hypersynchrony. CBZ and PHT may also depress ascending reticular excitatory inputs, resulting in hyperpolarization of thalamic neurons, oscillatory thalamocortical activity, and, in turn, spike-wave discharges. While valproic acid can also block sodium channels and increase the effects of GABA on postsynaptic GABA_A_ receptors, it does not typically cause ASE, as the GABA_A_ autoreceptor negative feedback circuit decreases GABA release [13].

Other studies have explored the typically good prognosis of ASE. Unlike CPSE in which N-methyl-D-aspartate mediates neuronal damage and neuron specific enolase (NSE) is released, no elevation of NSE levels is seen following ASE [12, 14]; this may explain the relatively good outcomes observed [12].

First-line acute treatment for ASE in pediatric patients is lorazepam (0.05–0.1 mg/kg IV). Alternatively, diazepam may be administered as initial monotherapy [7, 15]. If ASE persists, intravenous valproate is indicated as a second monotherapy following an initial trial of benzodiazepine [15]. In nonhospital settings, patients may be counselled to self-administer midazolam (buccal administration) or diazepam (rectal administration) at the onset of ASE [4]. Although the time frame during which ASE may cause long-term injury to or alteration of neuronal networks is currently unknown [3], aggressive treatment is generally not recommended [7]. However, the possibility of ASE evolving into a generalized tonic-clonic seizure if not effectively treated must also be carefully considered [7].

Long-term treatment of childhood absence epilepsy with ethosuximide or valproate is effective for over 80% of pediatric patients with remission usually occurring 2–5 years following onset [4]. Prognosis is usually excellent in typical ASE with underlying genetic epilepsy, though it is less favorable for atypical ASE with an underlying symptomatic or cryptogenic epilepsy syndrome [2].

## 4. Conclusion

Due to the subtle and variable manifestations of the condition, ASE may be underdiagnosed in children, particularly those not yet diagnosed with epilepsy. Awareness of this entity is critical and should be considered in the differential diagnosis for children with altered mental status. Clinicians should increase parental awareness of the signs and symptoms of ASE to facilitate early recognition and treatment and minimize risk of progression to generalized tonic-clonic seizures. Precipitating medications such as CBZ, PHT, TGB, and VGB should be avoided. Further research is required to determine the prevalence of ASE in pediatric patients, identify patients at increased risk of developing ASE, and determine any potential long-term sequelae of prolonged or recurrent ASE. With proper identification and early management, children with epilepsy syndromes with absences and ASE can lead more functional, seizure-free lives.

## References

[1] Genton P., Ferlazzo E., Thomas P. (2008). Absence status epilepsy: delineation of a distinct idiopathic generalized epilepsy syndrome. *Epilepsia*.

[2] Sutter R., Kaplan P. W. (2012). Electroencephalographic criteria for nonconvulsive status epilepticus: synopsis and comprehensive survey. *Epilepsia*.

[3] Trinka E., Cock H., Hesdorffer D. (2015). A definition and classification of status epilepticus—report of the ILAE Task Force on Classification of Status Epilepticus. *Epilepsia*.

[4] Panayiotopoulos C. P. (1999). Typical absence seizures and their treatment. *Archives of Disease in Childhood*.

[5] Panayiotopoulos C. P., Ferrie C. D., Koutroumanidis M., Rowlinson S., Sanders S. (2001). Idiopathic generalised epilepsy with phantom absences and absence status in a child. *Epileptic Disorders*.

[6] Rubboli G., Gardella E., Capovilla G. (2009). Idiopathic generalized epilepsy (IGE) syndromes in development: IGE with absences of early childhood, IGE with phantom absences, and perioral myoclonia with absences. *Epilepsia*.

[7] Korff C. M., Nordli D. R. (2007). Diagnosis and management of nonconvulsive status epilepticus in children. *Nature Clinical Practice Neurology*.

[8] Mahale R., Javali M., Mehta A., Sharma S., Madhusudhan B. K., Srinivasa R. (2015). Acute behavioral abnormality in an adolescent: absence status. *Pediatric Neurology*.

[9] Baykan B., Gökyiğit A., Gürses C., Eraksoy M. (2002). Recurrent absence status epilepticus: clinical and EEG characteristics. *Seizure*.

[10] Benson P. J., Klein E. J. (2001). New-onset absence status epilepsy presenting as altered mental status in a pediatric patient. *Annals of Emergency Medicine*.

[11] Thomas P., Valton L., Genton P. (2006). Absence and myoclonic status epilepticus precipitated by antiepileptic drugs in idiopathic generalized epilepsy. *Brain*.

[12] Hasan M., Lerman-Sagie T., Lev D., Watemberg N. (2006). Recurrent absence status epilepticus (spike-and-wave stupor) associated with lamotrigine therapy. *Journal of Child Neurology*.

[13] Osorio I., Reed R. C., Peltzer J. N. (2000). Refractory idiopathic absence status epilepticus: a probable paradoxical effect of phenytoin and carbamazepine. *Epilepsia*.

[14] Shirasaka Y. (2002). Lack of neuronal damage in atypical absence status epilepticus. *Epilepsia*.

[15] Wheless J. W., Clarke D. F., Carpenter D. (2005). Treatment of pediatric epilepsy: expert opinion, 2005. *Journal of Child Neurology*.

